# Functional Properties of *Brosimum alicastrum* Seed Flour Gels: Impact of Temperature, Concentration, and Additives on Rheology, Viscoelasticity, and Texture

**DOI:** 10.3390/ijms27198708

**Published:** 2026-09-29

**Authors:** Ángel H. Cabrera-Ramírez, Élida Gastélum-Martínez, Isabel Elizondo-De La Fuente, María D. García-Parra, Javier Jiménez-Hernández, Carla Arancibia, Manuel O. Ramírez-Sucre

**Affiliations:** 1 Centro de Investigación y Asistencia en Tecnología y Diseño del Estado de Jalisco, A.C., Int. Parque Científico Tecnológico de Yucatán s/n, Km 5.5 Carretera, Sierra Papacal-Chuburná, Chuburná, Mérida 97302, Yucatán, Mexico; acabrera@ciatej.mx (Á.H.C.-R.); egastelum@ciatej.mx (É.G.-M.); iselizondo_al@ciatej.edu.mx (I.E.-D.L.F.); mparra@ciatej.mx (M.D.G.-P.); 2 Centro de Innovación, Competitividad y Sostenibilidad (CICS), Universidad Autónoma de Guerrero, Acapulco 39640, Guerrero, Mexico; jjimenez@uagro.mx; 3 Departmento de Ciencia y Tecnología de Alimentos, Facultad Tecnológica, Universidad de Santiago de Chile, Obispo Umaña 050, Estación Central 9170201, Chile; carla.arancibia@usach.cl

**Keywords:** food hydrocolloids, gelling properties, thickening agent, starch–protein interactions, salt effects, ramón seed flour, *Brosimum alicastrum*, sustainable ingredients

## Abstract

Ramón seed flour (RSF), obtained from the drought- and hurricane-resistant *Brosimum alicastrum*, is a promising sustainable ingredient with high productivity and a favorable nutritional profile, but its functional properties remain poorly understood. This study characterized the rheological, viscoelastic, and textural behavior of RSF gels prepared at 7.7, 9, or 11% (*w*/*w*), heated at 80, 90, or 100 °C, and formulated with 2% salt, 5% sugar, or no additive. Rheological and viscoelastic properties were measured using a controlled-stress rheometer, and textural properties were evaluated using a single-cycle compression test. All systems showed pseudoplastic behavior, with flow behavior index values ranging from 0.42 to 0.68. The strongest gels were obtained at 11% RSF and 90 °C, showing the highest apparent viscosity (12.91 ± 0.85 Pa·s), storage modulus (308 ± 22 Pa), and hardness (1.05 ± 0.08 N). Salt had a temperature-dependent effect, increasing viscosity and hardness at 90 and 100 °C by 15–23%, but reducing them at 80 °C by 18–25%. In contrast, sugar consistently reduced viscosity, viscoelasticity, and hardness by 12–18%. These findings highlight the potential of RSF as a sustainable, plant-based thickener and underscore the importance of temperature–additive interactions on its functional performance.

## 1. Introduction

The global food system faces unprecedented challenges, including natural resource depletion, biodiversity loss, and climate change impacts that threaten agricultural productivity and food security [1,2]. In the Yucatan Peninsula of Mexico, deforestation for livestock and maize production has significantly altered land use patterns, with maize cultivation serving primarily as a starch source for the food industry [3]. The heavy reliance on a limited number of crop species, particularly maize, creates vulnerabilities in food supply chains and reduces agricultural resilience to climate variability [4]. Starch is the most widely used thickening agent in the food industry due to its abundance, low cost, and neutral taste [5]. However, the concentration of starch production in a few major crops (maize, wheat, potato, cassava) creates supply chain risks and limits opportunities for agricultural diversification. Exploring alternative starch sources from underutilized, climate-resilient species represents a strategic approach to enhancing food system sustainability while maintaining functional performance in food applications [6].

Starch gelatinization is a fundamental process in food manufacturing that determines the rheological and textural properties of numerous products. When starch granules are heated in the presence of water, they undergo irreversible structural changes including granular swelling, loss of crystalline order, and molecular solubilization [7,8]. This transformation creates a three-dimensional network that entraps water within a viscoelastic structure, modifying viscosity and texture [9]. The rheological behavior of starch-based systems is typically non-Newtonian and pseudoplastic, characterized by decreasing apparent viscosity with increasing shear rate [9]. This shear-thinning behavior is advantageous for food processing operations such as pumping, mixing, and swallowing. The viscoelastic properties of starch gels, quantified by storage modulus (G′) and loss modulus (G″), reflect the balance between elastic (solid-like) and viscous (liquid-like) behavior, with G′ > G″ indicating a gel network structure [9,10]. Recent studies have demonstrated that the functional properties of starch gels are highly sensitive to processing conditions and the presence of additives. Temperature profoundly affects gelatinization kinetics and the extent of starch granule disruption [7]. Salt and sugar, commonly present in food formulations, can significantly alter gelatinization temperature, pasting properties, and gel strength through mechanisms involving water activity modification, ionic interactions, and hydrogen bonding competition [11,12,13]. Notably, these additive effects are often concentration- and temperature-dependent, with some studies reporting biphasic or even opposite effects under different processing conditions [14].

*Brosimum alicastrum* Swartz (Moraceae), commonly known as ramón, breadnut, or Maya nut, is a multipurpose tree native to Mesoamerica that has sustained indigenous communities for millennia [15,16]. The species exhibits remarkable climate resilience, tolerating both drought and hurricane conditions while maintaining high seed productivity (28.6 tons/ha/year), substantially exceeding typical maize yields [17]. Despite these advantages, ramón remains underutilized in modern food systems, with seeds primarily relegated to livestock feed [15]. Ramón seeds possess a favorable nutritional profile that distinguishes them from conventional cereal starches. The seeds contain approximately 70% carbohydrates (63% starch), 11% protein, 13% dietary fiber, and significant levels of minerals (calcium, iron, zinc) and bioactive compounds including phenolic acids and flavonoids [18,19]. The protein content is notably higher than refined cereal starches, and the amino acid profile is rich in tryptophan, exceeding levels found in oats, beans, and maize [16,18]. This nutritional composition suggests that ramón seed flour (RSF) could provide functional benefits beyond simple thickening, potentially contributing to protein fortification and dietary fiber enrichment in food products.

Previous research on ramón has focused primarily on compositional characterization and ingredient substitution in baked goods. Studies have demonstrated that ramón flour can successfully replace up to 60% of wheat flour in cookies and 10–20% in tortillas, improving protein, fiber, and antioxidant content while maintaining acceptable sensory properties [20]. Consumption of ramón-fortified foods has been shown to improve nutritional status in elderly populations, increasing protein, fiber, and mineral intake while reducing cholesterol levels [19].

Despite growing interest in ramón as a functional food ingredient, critical knowledge gaps remain regarding its physicochemical behavior during processing. While basic starch characterization has been reported—including amylose content (25.36%), gelatinization peak temperature (~83 °C), gelatinization enthalpy (21.4 J/g), and swelling power (17.64 g/g at 90 °C)4—comprehensive rheological and viscoelastic characterization under processing-relevant conditions is lacking. No studies have systematically investigated the effects of concentration, temperature, and common food additives (salt, sugar) on RSF gel properties, nor have the interactions among these factors been explored. Understanding these structure–function relationships is essential for several reasons. First, rheological properties determine processing behavior (pumpability, mixability) and influence product texture and mouthfeel [9]. Second, the response to additives affects formulation flexibility and the ability to tailor functional properties for specific applications [6]. Third, temperature-dependent behavior informs optimal processing conditions and helps predict performance across different manufacturing scenarios [7]. Finally, comparative data relative to conventional thickeners are needed to assess RSF’s competitive potential and identify suitable application niches [21]. This study addresses these knowledge gaps by providing the first comprehensive characterization of RSF gel rheology, viscoelasticity, and texture as functions of concentration (7.7, 9, 11% *w*/*w*), processing temperature (80, 90, 100 °C), and additive type (salt, sugar, control). By systematically mapping RSF’s functional behavior, this research provides essential data to support its development as a sustainable, climate-resilient alternative to conventional thickening agents, contributing to both food system resilience and agricultural diversification in Mesoamerica.

## 2. Results and Discussion

### 2.1. Flow Behavior and Pseudoplastic Properties

#### General Flow Characteristics

**Figure 1:**
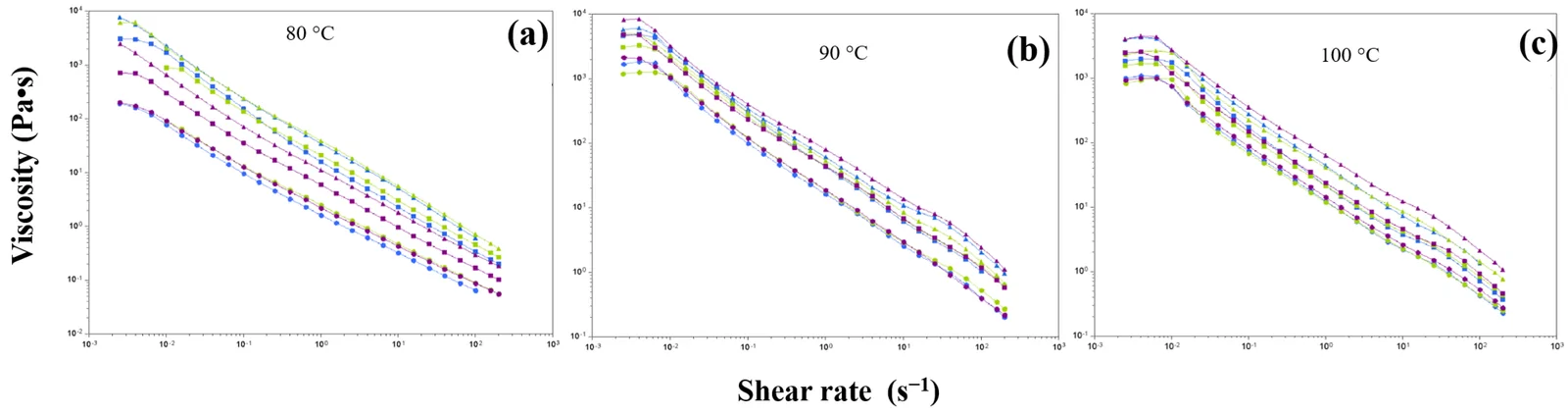
Flow curves of RSF gels processed at different temperatures: (**a**) 80 °C, (**b**) 90 °C, and (**c**) 100 °C. RSF concentrations were 11% (▲), 9% (■), and 7.7% (●). Treatments with sugar, salt, and the control are shown in green, purple, and blue, respectively.

The rheological parameters obtained from the Carreau model fitting revealed that all RSF systems exhibited pronounced pseudoplastic (shear-thinning) behavior (Figure 1), with flow behavior index (n) values ranging from 0.09 to 0.22 (Table 1). This behavior is characteristic of starch-based systems, in which structural organization is progressively disrupted and polymeric components become oriented under increasing shear, resulting in decreased resistance to flow [20,22]. Flow behavior indices below unity are consistent with the shear-thinning behavior commonly reported for gelatinized starch systems [20,22].

The concentration of RSF proved to be the factor with the greatest influence on the rheological properties, particularly on the zero-shear viscosity (η_0_). A direct relationship between concentration and η_0_ was observed, with increases of up to two orders of magnitude when the concentration rose from 7.7% to 11% *w*/*w*. This behavior can be attributed to the higher volumetric fraction of swollen starch granules and the increased density of crosslinks within the resulting three-dimensional network [7,23]. At higher RSF concentrations, greater amounts of amylose and amylopectin are available to establish intermolecular interactions through hydrogen bonding, leading to more rigid and viscous structures.

The relaxation time (λ) also increased significantly with concentration, reaching maximum values of 574.7 × 10^2^ s for the Control and 680.7 × 10^2^ s for the salt treatment, both at 80 °C and 11% concentration. These elevated λ values indicate that the structures formed exhibit greater elasticity and require more time to respond to applied deformation, a behavior characteristic of denser and more highly crosslinked polymeric networks [22].

Processing temperature exerted a complex influence on the rheological behavior, primarily mediated by the degree of starch gelatinization and the potential thermal degradation of the structures formed. At 80 °C—representing the lower boundary of the typical gelatinization range for legume starches (75–95 °C)—the highest values of η_0_ and λ were observed, particularly at elevated RSF concentrations. However, this condition also exhibited the greatest experimental variability (i.e., high standard deviations), as illustrated by the Control treatment at 11% (η_0_ = 849.9 ± 36.3 × 10^3^ Pa·s). Such variability suggests that gelatinization at 80 °C may be incomplete or heterogeneous, producing systems with differing proportions of partially swollen granules and fully gelatinized material. The coexistence of these structurally distinct phases contributes to the reduced reproducibility observed [6,24].

Treatments processed at 90 °C demonstrated a more favorable balance between structural development and reproducibility. At this temperature, gelatinization is presumably more complete, resulting in more homogeneous systems. Apparent viscosity at 10 s^−1^ (ηap) reached its maximum at 90 °C for several treatments (Sugar 11%: 9.95 ± 0.63 Pa·s; Salt 11%: 12.91 ± 1.19 Pa·s; Control 11%: 10.93 ± 0.18 Pa·s). These results indicate that 90 °C represents the optimal temperature for the development of rheological properties in RSF systems, consistent with previous reports for legume starches [12,25].

At the highest processing temperature evaluated (100 °C), a general trend toward lower η_0_ and λ values was observed, accompanied by an increase in η∞. This behavior can be attributed to several phenomena: partial thermal degradation of amylose and amylopectin chains; granule rupture with greater release of soluble material; and reduced retrogradation capacity during subsequent cooling. The higher η∞ values at 100 °C (Control 11%: 0.87 Pa·s vs. 0.20 Pa·s at 80 °C) indicate that, although the overall structure is less viscous, the dispersed phase contributes more substantially to the residual viscosity at high shear rates [8,26].

Regarding additive effects, the influence of sucrose (5% *w*/*w* in the final system) on the rheological response depended on both RSF concentration and processing temperature. At 11% RSF, for example, apparent viscosity was slightly lower than that of the control at 90 °C (9.95 ± 0.63 vs. 10.93 ± 0.18 Pa·s), whereas at 80 and 100 °C the sucrose-containing systems showed comparable or slightly higher values. Relaxation time also showed a condition-dependent response, being lower than the control at 80 °C but higher at 90 and 100 °C. These results indicate that the effect of sucrose on RSF rheology cannot be described as uniformly inhibitory within the conditions evaluated. Rather, sucrose appears to modulate starch hydration and network development in a manner dependent on the thermal and solids conditions of the system [11,12,27].

The behavior of salt-containing systems (2% *w*/*w* NaCl in the final system) showed a clear dependence on processing temperature. At 80 °C, η_0_ values were generally lower than those of the corresponding control at 7.7% and 11% RSF, suggesting that salt interfered with gelatinization at lower temperatures. At 90 °C, the rheological response increased markedly, with the highest apparent viscosity in Table 1 observed for the salt treatment at 11% RSF (12.91 ± 1.19 Pa·s). At 100 °C, the behavior remained stronger than or comparable to the control, indicating that once the thermal treatment was sufficient to overcome the salt-induced delay in gelatinization, the systems developed highly structured networks [11,13,14].

A notable finding was the exceptionally high relaxation time observed for the salt treatment at 80 °C and 11% RSF (λ = 680.7 ± 64.8 × 10^2^ s), the highest value recorded in the entire study. This indicates that when gelatinization occurs in the presence of salt at low temperature, the resulting structures exhibit pronounced elastic characteristics, possibly due to a distinct molecular conformation of the starch chains. Systems without additives exhibited the greatest gelation potential, with the highest η_0_ value recorded in the study (849.9 × 10^3^ Pa·s at 80 °C, 11%). However, they also showed the greatest variability, particularly at lower temperatures. The lowest flow behavior indices (*n* = 0.09 at 80 °C and 90 °C for 11%) indicate the most pronounced pseudoplastic character, associated with more complex and highly organized three-dimensional structures [28]. These results confirm that both sugar and salt act as gelation modifiers, reducing the structural complexity of the systems.

### 2.2. Viscoelastic Properties

#### 2.2.1. Gel Network Characterization

**Figure 2:**
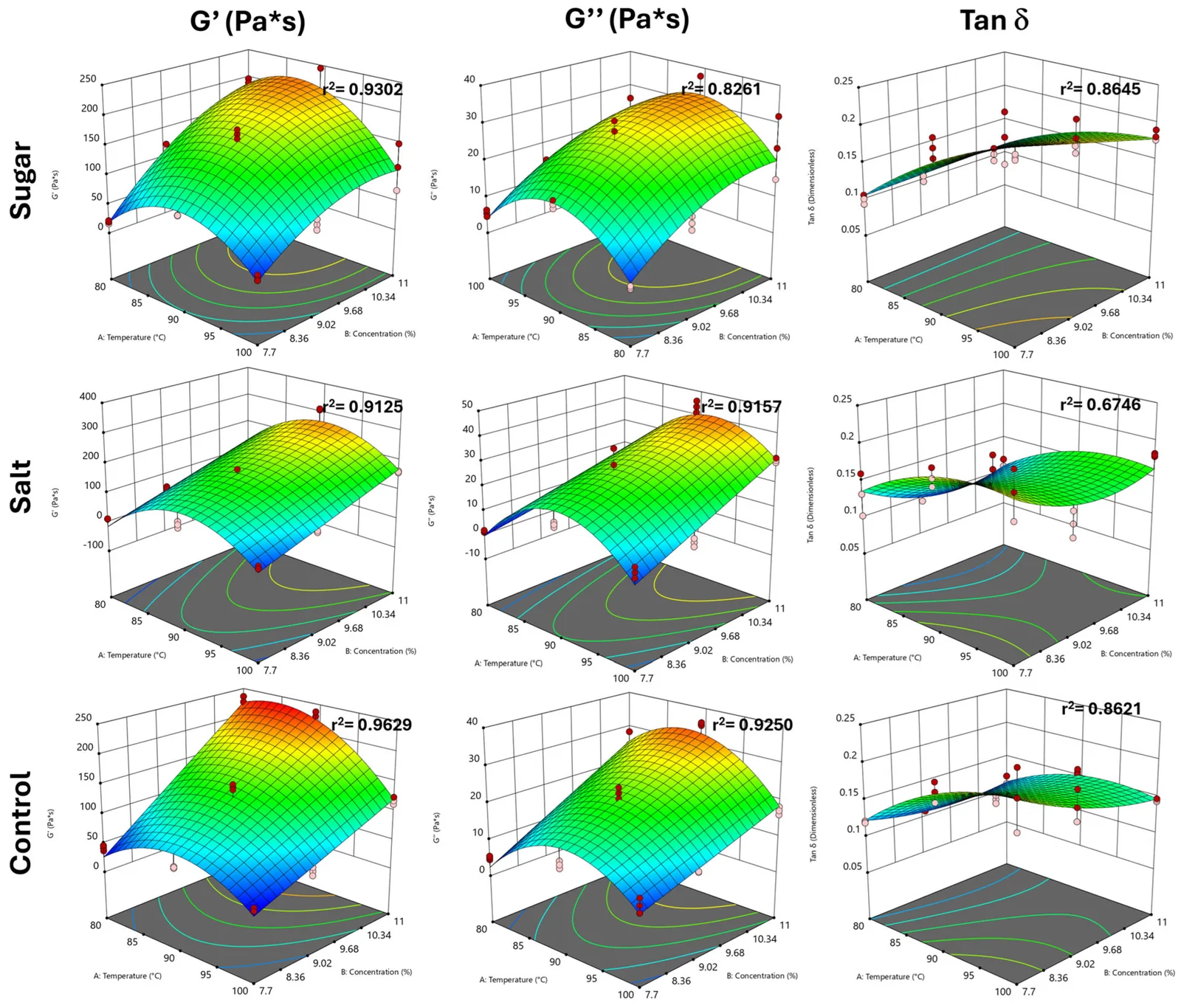
Response surface plots showing the combined effects of RSF concentration and processing temperature on the storage modulus (G′), loss modulus (G″), and loss tangent (tan δ) measured at 1 Hz for gels prepared with sugar, salt, or without additives (control). Surfaces illustrate the overall response trends for each additive condition. The circles represent experimental data points; differences in point shading indicate observations positioned above (darker point) or below (lighter point) the fitted response surface.

The flow analysis presented in Section 3.1 established that RSF dispersions behave as structured, shear-thinning systems. Building on that response, viscoelastic measurements were used to determine whether the same formulations also developed stable gel networks after cooling. As shown in Figure 2, all RSF systems exhibited predominantly elastic behavior, with G′ consistently exceeding G″ across the evaluated frequency range (0.1–10 Hz) and tan δ remaining below 1. This pattern indicates the formation of weak physical gel-like networks with predominantly elastic behavior, in which G′ exceeds G″ but the relatively low modulus values and tan δ values up to approximately 0.31 are consistent with limited network strength. Moreover, the weak frequency dependence of both moduli suggests the presence of relatively stable, weakly structured networks with limited relaxation over the time scale tested. The loss tangent (tan δ) remained below 1 across the evaluated treatments, confirming that the elastic contribution predominated over the viscous component. Although the magnitude of the viscoelastic response varied with concentration, temperature, and additive composition, the systems retained a predominantly elastic, weak gel-like character.

The pseudoplastic nature of RSF gels is advantageous for food applications, as it facilitates processing operations (pumping, mixing, filling) at high shear rates while providing desirable thickness and mouthfeel at the lower shear rates encountered during consumption [14]. Similar shear-thinning behavior has been reported for other alternative starch sources, including sacha inchi, taro, and pineapple stem starches [29,30], as well as for conventional maize and potato starch systems [7,21].

The effects of RSF concentration and processing temperature on the storage modulus (G′), loss modulus (G″), and loss tangent (tan δ), evaluated at 1 Hz, were visualized using response surface plots (Figure 2). This representation was selected to emphasize the overall response trends and the combined effects of concentration and temperature within each additive condition rather than individual point-to-point comparisons. All RSF systems exhibited predominantly elastic behavior [9], with storage modulus (G′) consistently exceeding loss modulus (G″) across the entire frequency range tested (0.1–10 Hz), resulting in loss tangent values below 1.

At 1 Hz, G′ values ranged from 45 ± 5 Pa (7.7%, 80 °C, sugar) to 308 ± 22 Pa (11%, 90 °C, control), while G″ values ranged from 12 ± 2 Pa to 78 ± 6 Pa for the same conditions. The G′/G″ ratio varied from 3.2 to 4.5, confirming the predominantly elastic character of the gels. These viscoelastic properties are comparable to those reported for other starch-based gel systems, including potato starch (G′ = 200–400 Pa at 10% concentration), cornstarch–methyl cellulose blends (G′ = 150–300 Pa), and rice starch–curdlan composites (G′ = 250–350 Pa).

#### 2.2.2. Concentration and Temperature Effects on Viscoelasticity

Factorial ANOVA showed that both concentration and temperature had highly significant effects on G′ and G″ (*p* < 0.001), with concentration being the dominant factor, while the response surfaces in Figure 2 illustrate the direction and magnitude of these effects. Increasing concentration from 7.7% to 11% resulted in clear increases in both moduli for the control samples heated to 90 °C, confirming that higher content of solids favored the development of relatively stronger gel-like networks. This concentration dependence is consistent with the increased density of junction zones and entanglement points as starch concentration rises.

A concentration-dependent increase in the viscoelastic moduli was also generally observed in the systems containing salt and sucrose, although the magnitude of this response depended on processing temperature and additive type (Figure 2). Salt-containing systems showed a marked temperature-dependent behavior: the viscoelastic response was comparatively weaker at 80 °C, particularly at lower RSF concentrations, whereas higher G′ and G″ values were observed as concentration increased at 90 and 100 °C. In contrast, sucrose-containing systems generally exhibited a more moderate increase in viscoelastic response with increasing RSF concentration, indicating that the effect of increasing the content of solids was modulated by the presence of sucrose. Thus, although increasing RSF concentration generally promoted greater elastic and viscous responses, the extent of network development depended on the interaction between the content of solids, processing temperature, and additive composition.

Temperature effects on viscoelasticity paralleled those observed for viscosity, with G′ and G″ generally increasing from 80 to 90 °C and then plateauing or decreasing slightly at 100 °C. This trend was most clearly observed in the control and salt-containing systems, whereas the sucrose-containing samples displayed a comparatively less pronounced temperature-dependent response. The higher elastic response observed around 90 °C aligns with the viscosity data and reinforces the conclusion that this temperature provides the best balance between complete gelatinization and preservation of network integrity. The slight decrease in G′ at 100 °C, particularly evident at higher concentrations, may result from greater granule disruption and molecular rearrangement, which could reduce the number of effective junction zones [7,8,24].

The loss tangent (tan δ) remained relatively constant across most treatments, with values generally indicating elastic-dominated but weak physical gel-like behavior. This consistency suggests that while the magnitude of both elastic and viscous responses increases with concentration and temperature, their relative contributions to overall viscoelastic behavior remain balanced [9].

#### 2.2.3. Additive Effects on Gel Network Strength

Salt and sugar effects on viscoelastic properties mirrored their impact on flow behavior. Salt exhibited temperature-dependent effects, weakening the elastic response at 80 °C but strengthening it at 90 and 100 °C for the most concentrated gels. This biphasic response reinforces the mechanistic interpretation proposed for viscosity: at sub-optimal temperatures, salt delays gelatinization and weakens network formation, whereas at higher temperatures, ionic interactions appear to strengthen interchain associations and enhance gel elasticity. By contrast, sugar consistently reduced viscoelastic strength across treatments, in agreement with its inhibitory effect on granule swelling and network development [11,12,13,14,27].

The practical implication of these findings is that salt can be strategically used to modulate gel strength in a temperature-dependent manner, while sugar provides a more predictable viscosity- and elasticity-reducing effect across processing conditions. For applications requiring relatively firmer, weak gel-like structures, such as spoonable or semi-solid food systems, combining high RSF concentration (11%) with processing at 90 °C and moderate salt addition (2%) produced the highest viscoelastic response among the conditions evaluated. Conversely, for applications requiring softer, less elastic textures (e.g., beverages, thin sauces), lower concentrations, moderate temperatures, and sugar addition can be employed.

### 2.3. Textural Properties

**Figure 3:**
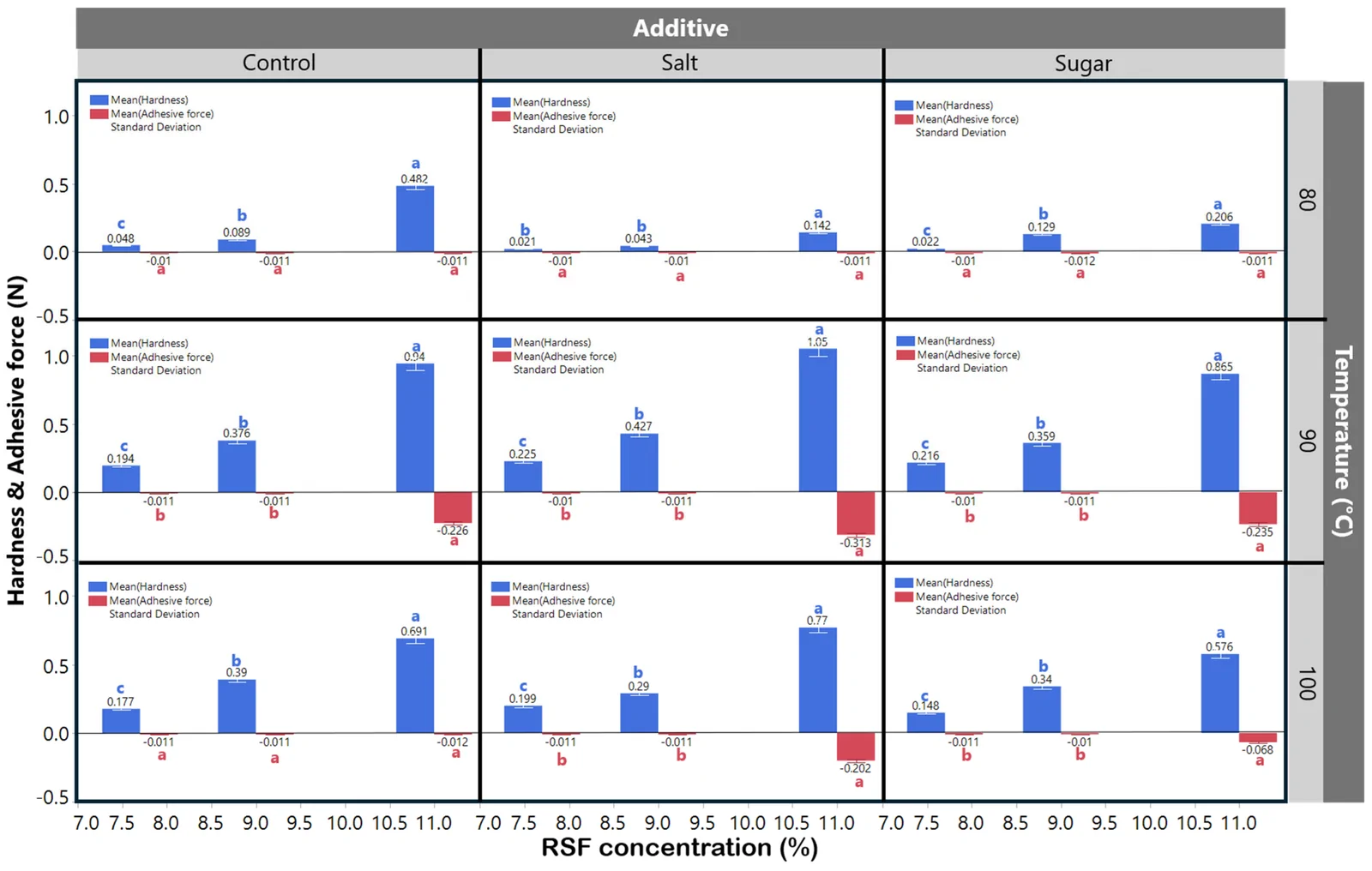
Textural properties of RSF gels as functions of concentration, temperature, and additive type. Bars represent standard deviation; different letters indicate statistically significant differences (*p* < 0.05) within each condition.

Whereas rheological and viscoelastic measurements describe the response of RSF gels under continuous and oscillatory deformation, texture analysis provides a more direct view of their macroscopic mechanical performance. In this context, hardness was the textural parameter most strongly affected by formulation and processing variables (Figure 3). At 7.7% and 9% RSF, hardness remained comparatively low and changed only modestly with additive treatment, indicating that at these concentrations the gel network was still limited in its capacity to resist compression. By contrast, at 11% RSF the effect of temperature became much more evident, with firmer gels developing mainly after processing at 90 and 100 °C. This trend is consistent with the rheological and viscoelastic results, which showed that higher solid content and near-complete gelatinization promoted stronger network formation. The comparatively weak texture observed at 80 °C further supports the interpretation that incomplete gelatinization restricts the establishment of a cohesive matrix. Overall, the texture data reinforce the view that RSF gel performance is governed by the combined effects of concentration, thermal treatment, and additive-induced modulation of water availability and network organization [9,24,31].

Adhesive force followed an inverse pattern relative to hardness, with more negative values generally observed under conditions that produced softer or less coherent gels. The most pronounced adhesiveness was recorded for the salt treatment at 11% RSF and 90 °C, indicating a matrix with greater surface stickiness despite its high solid content. This behavior suggests that not all highly concentrated systems formed equally cohesive structures; rather, the internal organization of the gel network determined whether the material behaved as a firm gel or as a more deformable and adhesive matrix. In this sense, the texture results complement the rheological findings by showing that high viscosity or solid content alone does not necessarily guarantee optimal mechanical integrity after cooling [16,31].

From an application standpoint, these results indicate that RSF functionality can be tuned over a relatively broad formulation window. Conditions around 11% RSF and 90 °C favored the development of stronger gels, particularly when the goal is to obtain structured systems with higher firmness. In contrast, lower concentrations or formulations containing sugar produced softer structures that may be more appropriate for fluid or semi-fluid applications. Taken together, the rheological, viscoelastic, and textural results converge on the same conclusion: the final performance of RSF is not determined by a single factor but by the interaction among concentration, thermal history, and additive composition. Because the texture analysis was based on a single compression cycle, parameters requiring a second compression cycle, such as cohesiveness and springiness, were not determined and should be considered in future studies for a more comprehensive characterization of RSF gel texture.

### 2.4. Relationship Among Rheological, Viscoelastic, and Textural Properties

**Figure 4:**
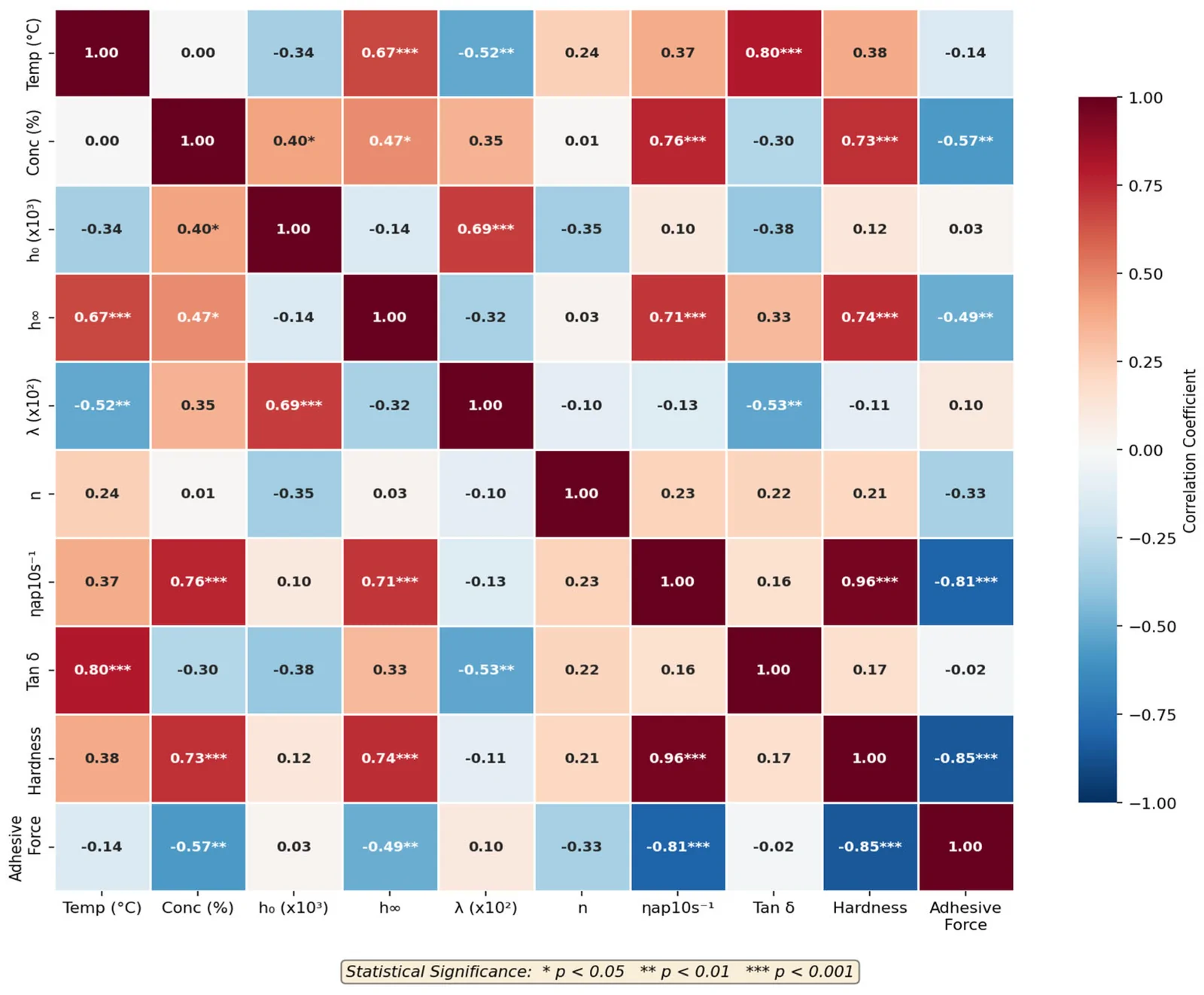
Pearson correlation among rheological parameters and textural properties of ramón seed flour (RSF) gels prepared under different processing conditions.

Pearson correlation analysis was performed to elucidate the relationships among rheological parameters and textural properties of Ramón seed flour (RSF) gels prepared under different processing conditions (Figure 4). The correlation matrix revealed several statistically significant relationships that provide insights into the structure–function relationships of RSF gel systems. The most prominent finding was the exceptionally strong positive correlation between apparent viscosity (ηap at 10 s^−1^) and gel hardness (r = 0.96, *p* < 0.001). This near-perfect correlation suggests that both parameters are governed by similar structural elements, presumably the density and strength of the starch gel network formed during gelatinization. Similar relationships have been reported for other starch-based systems, where increased network density simultaneously enhances resistance to flow and mechanical deformation [24,32]. Conversely, a strong negative correlation was observed between hardness and adhesive force (r = −0.85, *p* < 0.001), as well as between apparent viscosity and adhesive force (r = −0.81, *p* < 0.001). These inverse relationships indicate that firmer gels with more rigid network structures exhibit reduced surface adhesion, likely due to decreased molecular mobility at the gel surface and reduced ability to form intimate contact with adjacent surfaces. This finding has practical implications for food applications where specific textural attributes are desired.

#### Effect of Processing Variables on Gel Properties

The correlation analysis helped integrate the individual trends observed across the previous sections by showing how processing variables influenced multiple functional properties simultaneously. Temperature was positively correlated with tan δ (r = 0.80, *p* < 0.001) and η∞ (r = 0.67, *p* < 0.001), indicating that higher thermal treatment increased the viscous contribution to gel behavior and promoted greater flow under high shear. At the same time, temperature was negatively correlated with relaxation time (λ) (r = −0.52, *p* < 0.01), suggesting that gels formed at higher temperatures relaxed stress more rapidly, likely because of greater granule disruption and enhanced polymer mobility. RSF concentration showed strong positive correlations with apparent viscosity (r = 0.76, *p* < 0.001) and hardness (r = 0.73, *p* < 0.001), confirming that higher content of solids favored denser, mechanically stronger networks. Its negative correlation with adhesive force (r = −0.57, *p* < 0.01) further supports the idea that stronger gels tend to be less sticky and more structurally coherent.

A significant negative correlation was found between temperature and relaxation time (λ) (r = −0.52, *p* < 0.01), indicating that gels formed at higher temperatures exhibited faster stress relaxation. This behavior is consistent with more disrupted granular structures that allow for faster molecular rearrangement under applied stress. RSF concentration exhibited strong positive correlations with apparent viscosity (r = 0.76, *p* < 0.001) and hardness (r = 0.73, *p* < 0.001). These relationships confirm that increasing the solid content of RSF gels leads to denser network structures with enhanced mechanical properties. The negative correlation between concentration and adhesive force (r = −0.57, *p* < 0.01) further supports the interpretation that denser gel networks reduce surface adhesion characteristics.

The Carreau model parameters also revealed meaningful internal relationships that reinforce the structural interpretation of RSF gels. Zero-shear viscosity (η_0_) was strongly correlated with relaxation time (λ) (r = 0.69, *p* < 0.001), indicating that systems with greater resistance to flow also required longer times to adjust to deformation. Likewise, η∞ showed positive correlations with apparent viscosity (r = 0.71, *p* < 0.001) and hardness (r = 0.74, *p* < 0.001), suggesting that the high-shear response remained linked to overall network strength rather than to a purely soluble fraction. In contrast, the flow behavior index (n) was not significantly correlated with the other variables, implying that the degree of shear thinning was governed by structural features different from those controlling gel strength.

Collectively, these correlations showed that the same formulation factors that shaped flow behavior also determined viscoelasticity and final texture, thereby defining the practical functionality of RSF as a gel-forming ingredient. The trade-off between hardness and adhesive force suggests that formulators must balance these properties according to specific application requirements. Applications requiring firm, less sticky textures may benefit from higher RSF concentrations and processing around 90–100 °C, whereas applications requiring softer or more adhesive textures may be better served by lower concentrations or sugar-containing formulations.

## 3. Materials and Methods

### 3.1. Materials

Ramón (*Brosimum alicastrum* Swartz) seeds were collected from trees in the Yucatan Peninsula, Mexico, during the harvest season (May–July 2022). Seeds were cleaned, dried at 60 °C for 24 h, and ground using a hammer mill (Molinos Pulvex, Pulvex 200, CDMX, Mexico) to obtain flour with particle size < 250 μm (60 mesh). The flour was stored in sealed containers at 4 °C until use. Compositional analysis of the Ramón seed flour (RSF) (dry basis) revealed: 63.2 ± 1.5% starch, 11.3 ± 0.4% protein, 13.1 ± 0.6% dietary fiber, 5.8 ± 0.3% fat, 3.2 ± 0.2% ash, and 3.4 ± 0.3% moisture. Commercial-grade sodium chloride (NaCl, 99.5% purity) and sucrose (99.9% purity) were obtained from J.T. Baker (Phillipsburg, NJ, USA).

### 3.2. Preparation of RSF Gels

RSF gels were prepared at three concentrations (7.7, 9.0, and 11.0% *w*/*w*). These concentrations were selected based on preliminary experiments conducted in our laboratory, using the hydration characteristics of Ramón starch as a reference. Pérez-Pacheco et al. [33] reported a swelling power of 17.64 g water/g starch at 90 °C for starch isolated from *Brosimum alicastrum* seeds. Based on this hydration capacity, preliminary trials were carried out to establish flour-to-water ratios that provided sufficient water for starch granule hydration and swelling while generating systems with progressively increasing the contents of solids. From these preliminary trials, concentrations of 7.7, 9.0, and 11.0% (*w*/*w*) were selected because they produced workable dispersions spanning a range from less structured to more structured systems suitable for rheological and textural characterization. The flour was dispersed in distilled water with continuous stirring for 10 min at room temperature (25 ± 2 °C) to ensure complete hydration. For additive treatments, sodium chloride (2.0% *w*/*w*, final concentration) or sucrose (5.0% *w*/*w*, final concentration) was dissolved in the water prior to flour addition. These additive concentrations were selected based on typical levels used in food formulations: 2% NaCl represents a moderate salt level common in savory applications (soups, sauces, processed foods), while 5% sucrose is typical for mildly sweetened products (beverages, dairy desserts, some sauces) [5]. The dispersions were heated in a water bath (Thermo Scientific, TSGP02, Waltham, MA, USA) at a controlled heating rate of 5 °C/min to reach target temperatures of 80, 90, or 100 °C. Upon reaching the target temperature, samples were held for 10 min with gentle stirring (60 rpm) to ensure uniform heat distribution and complete gelatinization. The 10 min holding time was selected based on preliminary experiments showing that viscosity stabilized within this period, indicating completion of starch granule swelling and network formation. After heating, samples were cooled to 25 °C at a rate of approximately 2 °C/min in an ice-water bath and equilibrated for 30 min before rheological and textural measurements. All measurements were conducted at 25 °C to assess the properties of the fully formed gel network under standardized conditions relevant to storage and consumption.

The selection of heating temperatures (80, 90, 100 °C) was based on published gelatinization data for Ramón starch, which reports an onset temperature of approximately 75 °C, peak at 83 °C, and completion around 95 °C [26]. Thus, 80 °C represents partial gelatinization (early stage), 90 °C represents near-complete gelatinization (optimal stage), and 100 °C represents complete gelatinization with potential granule disruption (over-processing stage). This temperature range allows for the systematic exploration of gelatinization effects on functional properties.

### 3.3. Rheological Measurements

Rheological behavior was evaluated using a controlled-stress rheometer (TA Instruments, DHR2, New Castle, DE, USA) equipped with a 40 mm parallel-plate geometry and a 1050 μm gap, following the methodology previously described by Ramírez-Sucre and Baigts-Allende [34]. Measurements were performed at 25 ± 1 °C using a Peltier plate after a 5 min rest period to allow structural relaxation. Flow behavior was determined from viscosity–shear rate curves collected between 0.1 and 100 s^−1^. To describe the flow profiles, the experimental data were fitted to the Carreau–Yasuda model, which is widely used for starch- and hydrocolloid-based systems because it captures the low- and high-shear Newtonian regions as well as the intermediate shear-thinning domain. The storage modulus (G′), loss modulus (G″), and loss tangent (tan δ = G″/G′) were subsequently used to complement the flow analysis and are described in Section 2.4.

Model parameters included zero-shear viscosity (η_0_), infinite-shear viscosity (η∞), relaxation time (λ), and flow behavior index (n). Together, these parameters provided a quantitative basis for comparing the effects of concentration, temperature, and additives on RSF gel flow behavior and for linking the rheological response with the viscoelastic and textural results discussed below.

(1) $$\eta =\eta_{\infty }\alpha_{T}+\alpha_{T}\left({\eta_{0}-\eta }_{\infty }\right){\left[1+(\lambda \alpha_{T}\cdot {\dot{\gamma })}^{\alpha }\right]}^{\frac{n-1}{\alpha }}$$

where η_0_ and η∞ (Pa·s) represent the zero-shear and infinite-shear viscosities, respectively; λ (s) is a characteristic time constant, with its inverse being related to the characteristic shear rate for the onset of shear thinning; α is a dimensionless parameter governing the breadth of the transition from the Newtonian plateau to the power-law region; and n is the dimensionless flow behavior index describing the degree of shear-thinning behavior.

### 3.4. Viscoelastic Measurements

Viscoelastic properties were assessed using the same rheometer and geometry. Prior to frequency sweep tests, the linear viscoelastic region (LVR) was determined by conducting strain sweep experiments at a constant frequency of 1 Hz, varying strain amplitude from 0.01% to 10%. The LVR was defined as the strain range where G′ and G″ remained constant (variation < 5%). For all samples, the LVR extended to approximately 1% strain, and a strain of 0.5% (well within the LVR) was selected for subsequent frequency sweep measurements. Frequency sweep tests were conducted at 25 °C over a frequency range of 0.1 to 10 Hz at constant strain amplitude (0.5%). Storage modulus (G′, representing elastic behavior) and loss modulus (G″, representing viscous behavior) were recorded as functions of frequency. The loss tangent (tan δ = G″/G′) was calculated to quantify the relative contributions of viscous and elastic behavior. For comparative purposes, G′ and G″ values at 1 Hz were used for statistical analysis, as this frequency is commonly employed in food gel characterization [23].

### 3.5. Texture Analysis

The textural properties of RSF gels were evaluated using a Texture Analyzer (Shimatzu, EZ-SX, Kyoto, Japan) equipped with a 5 kg load cell and a cylindrical probe (P/0.5, 12.7 mm diameter). Gel samples were prepared in cylindrical molds (30 mm diameter × 20 mm height) and stored at 4 °C for 24 h to allow complete gel stabilization before testing. Prior to measurement, samples were equilibrated to 25 °C for 30 min. A single-cycle compression test was performed using the following parameters: pre-test speed 1.0 mm/s, test speed 1.0 mm/s, post-test speed 1.0 mm/s, compression distance 10 mm (50% strain), and a trigger force of 5 g. The following textural parameters were calculated from the force-time curve: hardness (N): maximum force during the compression cycle, representing gel firmness; adhesiveness force (N): maximum negative force recorded during probe withdrawal, representing the force required to overcome the attractive forces between the gel surface and the probe. Six replicate measurements were performed for each treatment, and mean values with standard deviations were reported.

### 3.6. Experimental Design and Statistical Analysis

A full factorial experimental design was employed with three factors, namely concentration (3 levels: 7.7, 9, 11% *w*/*w*), temperature (3 levels: 80, 90, 100 °C), and additive type (3 levels: control, 2% salt, 5% sugar), resulting in 27 treatment combinations. Each treatment was prepared in triplicate, and all measurements (rheological, viscoelastic, textural) were performed in triplicate for each preparation. Data were analyzed using three-way analysis of variance (ANOVA) to assess main effects and interactions. When significant effects were detected (*p* < 0.05), Tukey’s Honestly Significant Difference (HSD) post hoc test was applied for pairwise comparisons. Meanwhile, response surfaces were analyzed using Design-Expert software (version 12). Finally, a Pearson correlation was carried out to evaluate the relationship among evaluated variables. Statistical analyses were performed using Minitab 19 (Minitab LLC, State Collage, DE, USA). Results are presented as mean ± standard deviation, and differences were considered statistically significant at *p* < 0.05.

## 4. Conclusions

This study demonstrates that ramón seed flour (RSF) has promising functional performance as a gel-forming and thickening ingredient, with rheological, viscoelastic, and textural properties comparable to those of conventional starch-based systems. RSF dispersions exhibited consistent pseudoplastic behavior, while the highest relative gel strength and viscosity within the evaluated conditions were observed at 11% concentration and 90 °C, identifying processing conditions of particular relevance for food formulation. The results also revealed a marked temperature dependence in the effect of salt, which weakened gel structure at 80 °C but enhanced it at 90–100 °C, whereas it showed a condition-dependent effect on rheological behavior while generally reducing viscoelastic and textural responses. Together, these findings provide a clearer understanding of how concentration, temperature, and common additives govern RSF functionality.

Beyond its technological performance, RSF offers added value as a multifunctional ingredient due to its higher protein and fiber contents relative to refined starches, suggesting potential not only for thickening but also for nutritional enhancement. These attributes, combined with the climate resilience and agroecological relevance of *Brosimum alicastrum*, position RSF as a promising alternative for the development of more sustainable and diversified food systems. Although further work is needed to validate its behavior in complex food matrices, assess storage stability, clarify structure–function mechanisms, and quantify environmental and economic impacts, the present study provides the first robust foundation for its scientific and technological valorization. Overall, RSF emerges as a viable candidate for future food applications and as a strategic resource for advancing resilient, sustainable ingredient innovation in Mesoamerica and beyond.

## Figures and Tables

**Table 1:** Rheological parameters of flow curves adjusted to the Carreau–Yasuda model.

Additive	Temperature (°C)	Concentration (%)	η_0_ (×10^3^) (Pa × s)	η∞ (Pa × s)	λ (×10^2^) (s)	*n*	η_ap_	r^2^
Sugar	80	7.7	4.5 ± 1.3 ^B^	0.14 ± 0.17 ^BC^	134 ± 12.5 ^B^	0.22 ± 0.02 ^A^	0.51 ± 0.05 ^E^	0.9989
		9	3.9 ± 1.6 ^B^	0.03 ± 0.00 ^C^	5.3 ± 2.8 ^C^	0.15 ± 0.01 ^A^	2.83 ± 0.33 ^D^	0.9995
		11	276.1 ± 126.4 ^A^	0.04 ± 0.04 ^C^	321.5 ± 119.5 ^A^	0.14 ± 0.01 ^A^	5.69 ± 0.14 B^C^	0.9994
	90	7.7	1.4 ± 0.2 ^B^	0.22 ± 0.08 ^ABC^	1.9 ± 0.4 ^C^	0.16 ± 0.04 ^A^	2.85 ± 0.07 ^D^	0.9987
		9	4.4 ± 0.4 ^B^	0.36 ± 0.04 ^ABC^	2.5 ± 0.0 ^C^	0.14 ± 0.04 ^A^	5.91 ± 1.14 ^B^	0.9987
		11	10.7 ± 1.8 ^B^	0.22 ± 0.19 ^ABC^	9.2 ± 4.2 ^C^	0.22 ± 0.04 ^A^	9.95 ± 0.63 ^A^	0.9984
	100	7.7	1.1 ± 0.2 ^B^	0.27 ± 0.01 ^ABC^	2.3 ± 0.1 ^C^	0.17 ± 0.01 ^A^	2.16 ± 0.14 ^D^	0.9966
		9	1.7 ± 0.7 ^B^	0.44 ± 0.23 ^AB^	2.3 ± 0.3 ^C^	0.21 ± 0.07 ^A^	4.4 ± 0.29 ^C^	0.9966
		11	5.9 ± 3.8 ^B^	0.57 ± 0.20 ^AB^	5.6 ± 5.0 ^C^	0.21 ± 0.03 ^A^	9.12 ± 0.66 ^A^	0.9948
Salt	80	7.7	0.4 ± 0.0 ^B^	0.03 ± 0.00 ^C^	6 ± 0.1 ^C^	0.2 ± 0.02 ^A^	0.41 ± 0.03 ^F^	0.9987
		9	2.2 ± 0.8 ^B^	0.06 ± 0.01 ^C^	7.2 ± 1.4 ^C^	0.12 ± 0.05 ^A^	1.04 ± 0.11 ^EF^	0.9985
		11	118.6 ± 82.3 ^A^	0.08 ± 0.07 ^C^	680.7 ± 64.8 ^A^	0.17 ± 0.07 ^A^	1.81 ± 0.01 ^DE^	0.9992
	90	7.7	3.2 ± 0.5 ^B^	0.01 ± 0.02 ^C^	5.1 ± 0.2 ^C^	0.17 ± 0.02 ^A^	2.94 ± 0.07 ^D^	0.9979
		9	59.2 ± 7.5 ^AB^	0.19 ± 0.06 ^BC^	89.7 ± 11.77 ^B^	0.21 ± 0.02 ^A^	6.6 ± 0.47 ^B^	0.9991
		11	137.6 ± 16.3 ^A^	0.56 ± 0.44 ^AB^	144.2 ± 18.5 ^B^	0.21 ± 0.00 ^A^	12.91 ± 1.19 ^A^	0.9989
	100	7.7	1.4 ± 0.2 ^B^	0.3 ± 0.02 ^BC^	2.4 ± 0.0 ^C^	0.16 ± 0.04 ^A^	2.4 ± 0.33 ^D^	0.9973
		9	3.9 ± 0.4 ^B^	0.62 ± 0.05 ^AB^	3.6 ± 1.7 ^C^	0.15 ± 0.03 ^A^	4.71 ± 0.16 ^C^	0.9974
		11	5.9 ± 0.2 ^B^	0.77 ± 0.09 ^A^	3.6 ± 0.8 ^C^	0.22 ± 0.02 ^A^	12.19 ± 0.42 ^A^	0.9973
Control	80	7.7	10.1 ± 10.5 ^B^	0.04 ± 0.01 ^D^	344.6 ± 35.5 ^B^	0.19 ± 0.01 ^A^	0.37 ± 0.06 ^G^	0.9980
		9	3.1 ± 1.7 ^B^	0.13 ± 0.08 ^D^	3 ± 0.2 ^C^	0.09 ± 0.08 ^A^	2.22 ± 0.12 ^F^	0.9956
		11	849.9 ± 36.3 ^A^	0.2 ± 0.22 ^CD^	574.7 ± 11.2 ^A^	0.09 ± 0.04 ^A^	5.25 ± 0.20 ^CD^	0.9982
	90	7.7	2.8 ± 0.1 ^B^	0.18 ± 0.04 ^CD^	3.4 ± 1.2 ^C^	0.13 ± 0.03 ^A^	2.75 ± 0.24 ^EF^	0.9972
		9	13.6 ± 10.1 ^B^	0.4 ± 0.06 ^CD^	10.7 ± 11.1 ^C^	0.14 ± 0.03 ^A^	6.19 ± 0.03 ^C^	0.9984
		11	9.4 ± 2.5 ^B^	1 ± 0.28 ^A^	4.9 ± 1.9 ^C^	0.11 ± 0.13 ^A^	10.93 ± 0.18 ^A^	0.9972
	100	7.7	1.4 ± 0.1 ^B^	0.32 ± 0.10 ^CD^	2.6 ± 0.7 ^C^	0.15 ± 0.03 ^A^	2.22 ± 0.02 ^F^	0.9967
		9	2.3 ± 0.2 ^B^	0.56 ± 0.10 ^BC^	2.6 ± 1.4 ^C^	0.14 ± 0.08 ^A^	3.78 ± 0.08 ^DE^	0.9962
		11	5.5 ± 0.4 ^B^	0.87 ± 0.07 ^AB^	3.2 ± 0.3 ^C^	0.16 ± 0.03 ^A^	8.36 ± 1.51 ^B^	0.9963

η_0_: zero rate viscosity; η∞: infinite viscosity; λ: relaxation time; η_ap_ = apparent viscosity; *n* = flow behavior. Values are presented as mean ± standard deviation. Different superscript letters within each rheological parameter and additive indicate significant differences among temperature × concentration treatment combinations according to Tukey’s HSD test (*p* < 0.05), following a significant temperature × concentration interaction (*p* < 0.05).

## Data Availability

No new datasets were generated or analyzed during the current study. The original contributions presented in this study are included in the article. Further inquiries can be directed to the corresponding author.
